# The effects of beta-hydroxy-beta-methyl butyrate supplementation in surgical patients: a systematic review and meta-analysis of randomized controlled trials

**DOI:** 10.3389/fnut.2025.1621206

**Published:** 2025-07-23

**Authors:** Yan-Ge Hu, Ji-Heng Shi, Da-Xing Yu, Hui-Bin Huang

**Affiliations:** Department of Emergency, Fuxing Hospital of Capital Medical University, Beijing, China

**Keywords:** beta-hydroxy-beta-methyl butyrate, surgery, muscle mass, complication, meta-analysis

## Abstract

**Background:**

Beta-hydroxy-beta-methylbutyrate (HMB) is a nutritional supplement that has demonstrated favorable effects on restoring muscle mass. However, evidence to support its use in patients underlying surgery remains unclear. We aimed to conduct a systematic review and meta-analysis of HMB in this population to ascertain its effect.

**Methods:**

We searched PubMed, EMBASE, Web of Science, the China National Knowledge Infrastructure, Wanfang, and the Cochrane Library for randomized controlled trials (RCTs) focused on surgical patients receiving HMB compared to controls. The last search was March 15, 2025. Length of stay (LOS) and postoperative complications were the primary outcomes. We assessed study quality and performed subgroup analysis, sensitivity analysis, and the GRADE system to explore potential heterogeneity.

**Results:**

Eleven RCTs with 575 patients were included. There are some differences in study design, HMB protocols, and muscle measurements among these trials. Overall, HMB significantly reduced the hospital LOS (MD −0.90 days; 95% CI, −1.79 to −0.01; *I*^2^ = 0%, *p* = 0.05) and postoperative complications (RR 0.50; 95% CI, 0.32 to 0.79; *I*^2^ = 0%, *p* = 0.003). These findings were confirmed in most subgroup and sensitivity analyses. As to muscle measurements, the HMB group had significantly more mid-arm muscle-circumference (*p* = 0.05), appendix skeletal muscle mass (*p* = 0.03) and 6-min walking distances (*p* = 0.007), but had similar changes in skeletal muscle mass and lean body mass. Regarding nutritional status, compared to the control group, the HMB group did not show significant improvement from baseline after treatment, including body weight, body mass index, serum albumin, and total albumin (*p*-values from 0.10 to 0.63).

**Conclusion:**

HMB supplement seems to significantly improve hospital LOS and postoperative complications, as well as some outcomes of muscle measurements and physical function. However, due to the significantly heterogeneity among the included studies, more well-designed RCTs are needed to confirm our findings.

## Introduction

Recently, the nutritional status of surgical patients has gained significant attention (1). It is known that inadequate nutrition can negatively impact postoperative outcomes (2, 3). Specifically, protein-energy malnutrition and deficient in micronutrients and essential nutrients can increase inflammation, weaken immune function, and hinder wound healing (4). These issues can lead to decreased muscle mass, delayed recovery, and prolonged hospital stays (5). Research has shown that surgery-related muscle loss (SRML) is quite common, affecting about 38 to 52% of patients following major abdominal surgery (6, 7). Muscle wasting occurs due to various factors, such as chronic obstructive pulmonary disease, diabetes, older age, open resection operation, insufficient protein intake before surgery, and decreased physical activity following surgery (6, 8, 9). Studies indicate that patients experiencing SRML have more postoperative complications, and those who suffer from both loss of muscle quantity and quality loss have poorer overall survival rates compared to other groups (6–8). On the other hand, increased protein intake before surgery is associated with a lower risk of developing SRML. Despite this knowledge, current strategies to prevent muscle wasting, such as infection control, enhanced protein supplementation, and pharmacological treatments, have largely fallen short of effectiveness.

In recent years, research has highlighted the role of beta-hydroxy-beta-methylbutyrate (HMB) in maintaining skeletal muscle mass (10). HMB, a metabolite derived from leucine, is essential for muscle protein synthesis and helps reduce protein breakdown (11). Various studies have shown that HMB can mitigate muscle loss, maintain muscle strength and function in older adults, and aid in recovery from exercise-induced muscle injuries (12, 13). Consequently, HMB has gained considerable clinical interest. Several meta-analyses suggested that individuals suffering from sarcopenia (14), malnutrition (15), or cancer (16) may benefit from HMB supplementation, including increased muscle mass and strength. On the contrary, the benefits of HMB have not been consistently observed in critically ill patients, likely due to the highly heterogeneous nature of this population (17). Despite these findings, there is still a lack of comprehensive evidence from meta-analyses regarding the effectiveness of HMB in improving muscle mass and clinical outcomes, particularly in surgical patients.

Recently, several studies have been published on HMB supplementation in surgical patients (18–20). Therefore, with the strengths of meta-analysis, we aim to conduct a systematic review and meta-analysis to explore whether HMB supplementation could be beneficial to surgical patients in terms of clinically important outcomes and muscle maintenance.

## Methods

### Protocol

This systematic review and meta-analysis followed the PRISMA with 2020 updates and Cochrane Collaboration guidelines (Supplementary File 1) (21) by a pre-registered protocol (INPLASY202530123).

### Search strategy and selection criteria

Two authors (Y-GH and J-HS) performed a comprehensive search independently from inception until March 15, 2025, using PubMed, EMBASE, Web of Science, the China National Knowledge Infrastructure, Wanfang, and Cochrane Library. The search incorporated medical subject headings and keywords, specifically targeting terms like “β-hydroxy-beta-methylbutyrate” OR “beta-hydroxy-beta-methylbutyrate” OR “hydroxy methylbutyrate” AND “surgery” OR “operation” OR “operative,” without language or year restrictions. The search strategy is detailed in Supplementary File 2. Moreover, grey literature was explored through https://scholar.google.com and https://www.basesearch.net, and references of selected articles were examined for any eligible studies.

### Selection criteria

We included studies in the meta-analysis based on the following criteria. First, participants were adult patients over 18 years old undergoing surgery. Second, the intervention involved HMB in the experimental group, with no limitations on the dosage, administration route, duration of treatment, or use of additional supplements. Third, comparators were non-HMB interventions, placebo or conventional therapy. Fourth, only RCTs were included. Finally, the outcomes measured included clinical outcomes, muscle measurements, and nutritional status indicators. We excluded studies based on the following criteria: children or pregnant women, duplicate publications, or those designed as cohort studies, abstracts, reviews, or comments.

### Data extraction and outcomes

The two authors extracted relevant data from the tables, figures, texts or additional files from the included RCTs. These variables included trial characteristics (first author’s name, year of publication, country, and study design), patient characteristics (age, sex ratio, patient population, body mass index, and body weight), HMB and control regimens, and predefined outcomes. We preferred to use the intention-to-treat results of the included RCTs. For studies that provide results from assessments at different time points, we selected the longest assessment time points after treatment for inclusion in the meta-analysis. Any disagreements were resolved by consulting a third researcher (H-BH).

The primary outcomes were clinical indicators such as the length of stay (LOS) in the hospital and postoperative complications. Secondary outcomes included muscle measures [i.e., appendicular skeletal muscle mass (ASMM), mid-arm muscle-circumference (MAMC), lean body mass (LBM)], physical function [i.e., hand grip strength (HGS), gait speed, or 6-min walking distance (6-MWD)], and nutritional status [i.e., serum albumin, total albumin, body mass index (BMI), or body weight (BW)].

### Quality assessment

Y-GH and J-HS independently conducted quality assessments of each publication using the Cochrane Risk of Bias tool (version 2) (22). Publication bias was evaluated by visual inspection of funnel plots when 10 or more trials were available. We used the Grading of Recommendations Assessment, Development, and Evaluation (GRADE) system to evaluate the quality of evidence (23). The disagreements between the two authors were resolved by consulting a third author (H-BH).

### Statistical analysis

We used RevMan 5.4 software, as recommended by the Cochrane Library, for the meta-analysis. The mean differences (MD) or odds ratios (ORs) and their corresponding 95% confidence intervals (CIs) were used to assess efficacy. For studies reporting median and interquartile range (IQR) but not SD, we estimated the mean and SD from the median and IQR, respectively (24). We conducted meta-analyses on predefined outcomes when at least two trials were available for pooling. We used the *I*^2^ statistic to test for heterogeneity, with values of *I*^2^ < 50% and *I*^2^ > 50% indicating low and high heterogeneity, respectively. A fixed-effect model was used when *I*^2^ < 50%, and a random-effect model was used when *I*^2^ > 50% (25).

To test the outcomes’ robustness and explore the potential influence factors, we conducted sensitivity analyses to identify each study’s influence on the overall pooled estimate of the outcome of interest. We also performed subgroup analyses based on the following criteria: (1) exercise (with or without), (2) location (Asia or non-Asia), (3) patient age (≥65 years or <65 years), (4) HMB regimen (use of HMB alone or combined HMB with additional supplements), and (5) study design (double-blind or undouble-blind).

## Results

### Searching results

**Figure 1:**
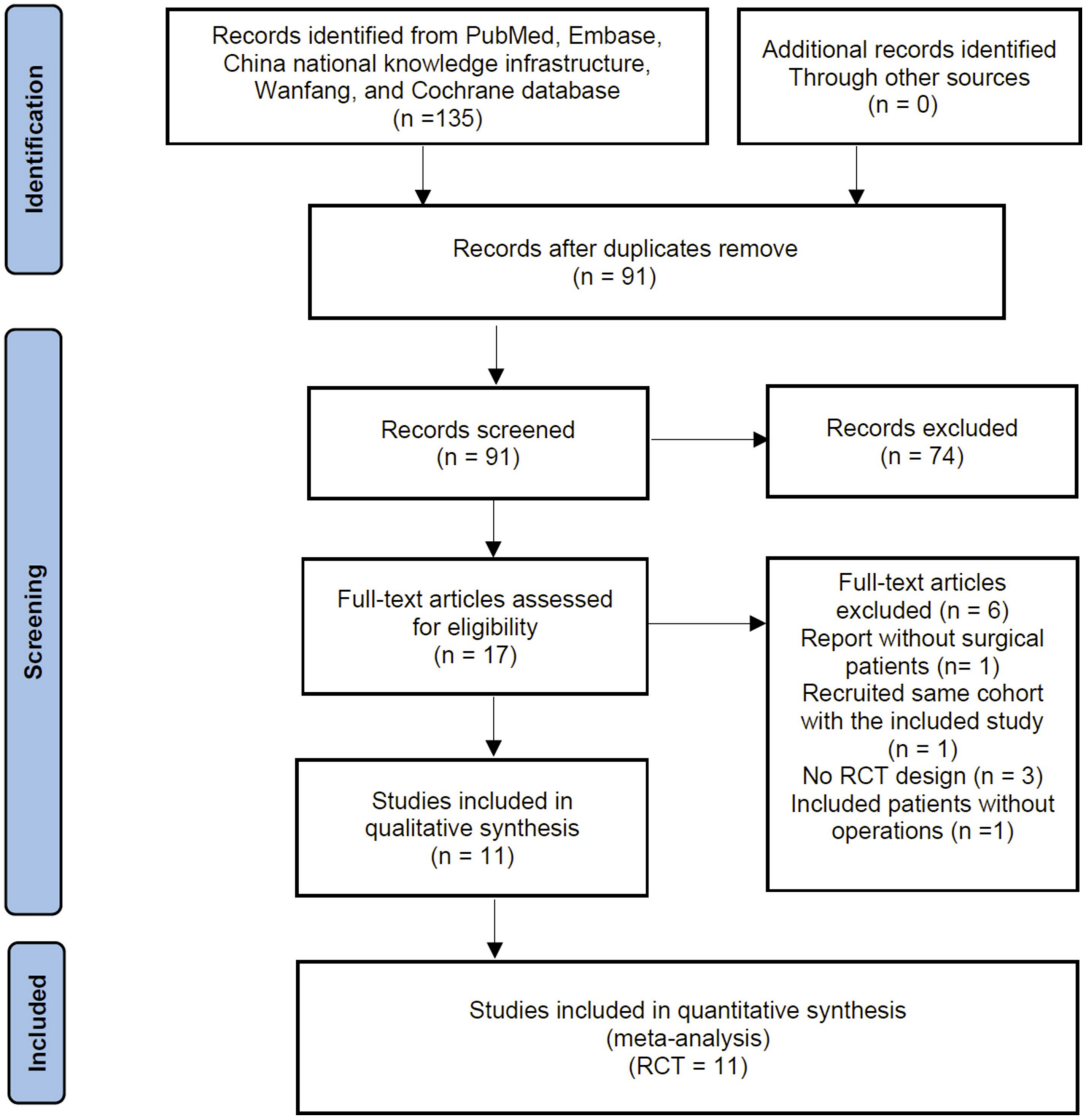
Flow chart of literature selection.

The primary search identified 135 records from the databases and additional searches. After removing duplicates, 91 records remained for title and abstract screening, of which 74 were excluded as they did not meet the inclusion criteria. A Subsequent full-text screening ruled out 6 RCTs, with the reasons for exclusion detailed in Supplementary File 3. Finally, 11 RCTs were included in the quantitative analyses (18–20, 26–33). The process of identification, screening and study inclusion is illustrated as a PRISMA flow diagram in Figure 1.

### Study characteristics

Table 1 summarizes the characteristics of the included RCTs. These trials, published from 2011 to 2025, were conducted in seven countries: Türkiye, China, Italy, Spain, Japan, Iran, and the United States. In total, 575 patients were analyzed, with 294 in the HMB group and 281 in the control group. Of these trials, nine were single-center (18–20, 26–29, 32, 33), while two were multi-center (30, 31), focusing on conditions like hip fracture (27, 28, 31), cardiac surgery (19, 20), cancer (30, 33), liver transplantation (18, 29), and endoscopic surgery (26). Five of the included RCTs administered HMB as a single supplement (20, 27, 29–31), while the other six combined HMB with arginine and glutamine (18, 19, 26, 28, 32, 33). All studies administered a daily dose of 3 g of HMB, taken as 1.5 g twice daily. Additionally, four trials incorporated exercises with HMB interventions (18, 20, 31, 32). Follow-up assessments were conducted in all RCTs, with the timing of outcome assessment ranging from 10 days to 12 months post-intervention. The details regarding the study strategies are summarized in Table 2. A total of six studies (18–20, 27, 30, 33) described the complications which were summarized in Supplementary File 6.

**Table 1:** Characteristics of the included studies.

Study	Country	Population	Design	*N*		Age (year)		Gender (%)		Primary outcome	Risk of bias
				HMB	Ctrl	HMB	Ctrl	HMB	Ctrl		
Clements et al. (26)	United States	LGB	SC, UB	14	16	47.9	46	0	6.2	Muscle measure	High
Ekinci et al. (28)	Türkiye	Hip fracture	SC, UB	38	37	82.2	83.1	0	0	Complications	High
Kamo et al. (18)	Japan	Liver transplantation	SC, DB	13	13	58.5	60	41.7	58.3	Grip strength	low
Lattanzi et al. (29)	Italy	Liver transplantation	SC, UB	12	10	60.4	59.3	100	100	Muscle measure	High
Malafarina et al. (31)	Spain	Hip fracture	MC, UB	55	52	85.7	84.7	32.7	18.6	Nutritional status	High
Nishizaki et al. (32)	Japan	Knee arthroplasty	SC, UB	13	10	71.1	69.8	60	62.8	Muscle measure	Unclear
Norouzi et al. (19)	Iran	Heart surgery	SC, DB	35	35	59	55	70	50	Myocardial biomarkers	Low
Ogawa et al. (20)	Japan	Cardiac surgery	SC, SB	22	22	71.8	72.5	68	64	Six-minute walking distance	Unclear
Wada et al. (33)	Japan	Malignancies	SC, DB	31	30	66	69	60	55.9	Complications	Low
Yang et al. (30)	China	Colon cancer	MC, UB	31	28	70.1	70.8	51.6	53.6	Nutritional status	Unclear
Zuo et al. (27)	China	Hip fracture	SC, UB	30	28	70.2	59.3	60	54.5	Nutritional status	Unclear

Ctrl, control; DB, double-blind; HMB, beta-hydroxy-beta-methyl butyrate; LGB, laparoscopic gastric bypass; MC, multicentre; SB, single-blind; SC, single-center; UB, unblind.

**Table 2:** Study strategies of the included RCTs.

Study	Timing of HMB administration	Nutrition protocol calories; protein	Intervention group	Control group	Exercise	Timing of evaluation	Muscle measure
Clements et al. (26)	Starting the day after surgery	Measured by IC	C-HMB 3 g (2 × 1.5-g doses/day) for 8 weeks; *n* = 14	Usual care; *n* = 16	NR	2, 8^b^ weeks PO	DXA
Ekinci et al. (28)	After surgery	Guided by a dietitian	C-HMB 3 g (2 × 1.5-g doses/day) for 30 days; *n* = 32	Usual care; *n* = 30	NR	15, 30^b^ days PO	None
Kamo et al. (18)	After surgery	From 10–15 to 25–30 kcal/kg/day; 1.2–1.5 g/kg/day	C-HMB 3 g (2 × 1.5-g doses/day) for 30 days; *n* = 12	Placebo; *n* = 11	ER	1, 2^b^ months PO	CT
Lattanzi et al. (29)	30 days after surgery	25–30 kcal/kg/day;1.2 g/kg/day	HMB 3 g (2 × 1.5-g doses/day) for 12 weeks; *n* = 12	Usual care; *n* = 10	NR	End of treatment, 6, 12^b^ months	DXA
Malafarina et al. (31)	Starting at the rehabilitation	1,500 kcal/day;87.4 g/day	HMB 3 g (2 × 1.5-g doses/day) until discharge; *n* = 49	Usual care; *n* = 43	ER	Discharge^b^	BIA
Nishizaki et al. (32)	Five days before surgery	NR	C-HMB 3 g (2 × 1.5-g doses/day) for 33 days; *n* = 13	Usual care; *n* = 10	ER	18,14 BO, and PO^b^	CT
Norouzi et al. (19)	30 days before cardiac surgery	NR	C-HMB 3 g (2 × 1.5-g doses/day) for 30 days; *n* = 30	Placebo; *n* = 30	NR	10 days PO^b^	None
Ogawa et al. (20)	At least 2 weeks before surgery	Dietary intake was guided by a dietitian	C-HMB 3 g (2 × 1.5-g doses/day) for at least 14 days; *n* = 22	Usual care; *n* = 44	ER	One day BO, and 2 weeks PO^b^	BIA
Wada et al. (33)	Once daily for 3 days preoperatively	NR	C-HMB 3 g (2 × 1.5-g doses/day) for 10 days; *n* = 30	Placebo; *n* = 30	NR	Discharge^b^	BIA
Yang et al. (30)	10 days before surgery	NR	HMB 3 g (2 × 1.5-g doses/day) for 40 days; *n* = 31	Usual care; *n* = 28	NR	30 days PO^b^	None
Zuo et al. (27)	At randomization	3-Phase nutrition program^a^	HMB 3 g (2 × 1.5-g doses/day) for 6 weeks; *n* = 30	Usual care; *n* = 28	NR	3, 6^b^ weeks PO	BIA

BIA, bioelectrical impedance analysis; BO, before operation; C-HMB, HMB combined with other supplements, such as arginine, or glutamine; CT, computed tomography; DXA, dual-emission X-ray absorptiometry; ER, early rehabilitation; HMB, beta-hydroxy-beta-methyl butyrate; IC, indirect calorimetry; NR, not report; PO, postoperative.

a The total daily calorie and protein supply at the three time stages (0–2 weeks, 2–4 weeks, and 4 weeks) was 1,400 kcal/day, 84 g/day; 1,650 kcal, 82 g/day; and 1,925 kcal/, 86 g/day, respectively.

b The results in this timing of evaluation were selected for inclusion in the meta-analysis.

### Quality assessment

The results of the risk of bias assessment of the included RCTs are presented in Supplementary File 4. The risk of bias in RCTs ranged from low to high in all critical domains. Evaluation of publication bias by visually inspecting funnel plots showed potential publication bias in the included trials (Supplementary File 5). Through the GRADE method, we rated the evidence for pooled data for hospital LOS, complication, HG, BW, BMI, ASMM, albumin, and 6-MWD as moderate, moderate, low, very low, very low, very low, and low, respectively (Supplementary File 6).

### Primary outcome

**Figure 2:**
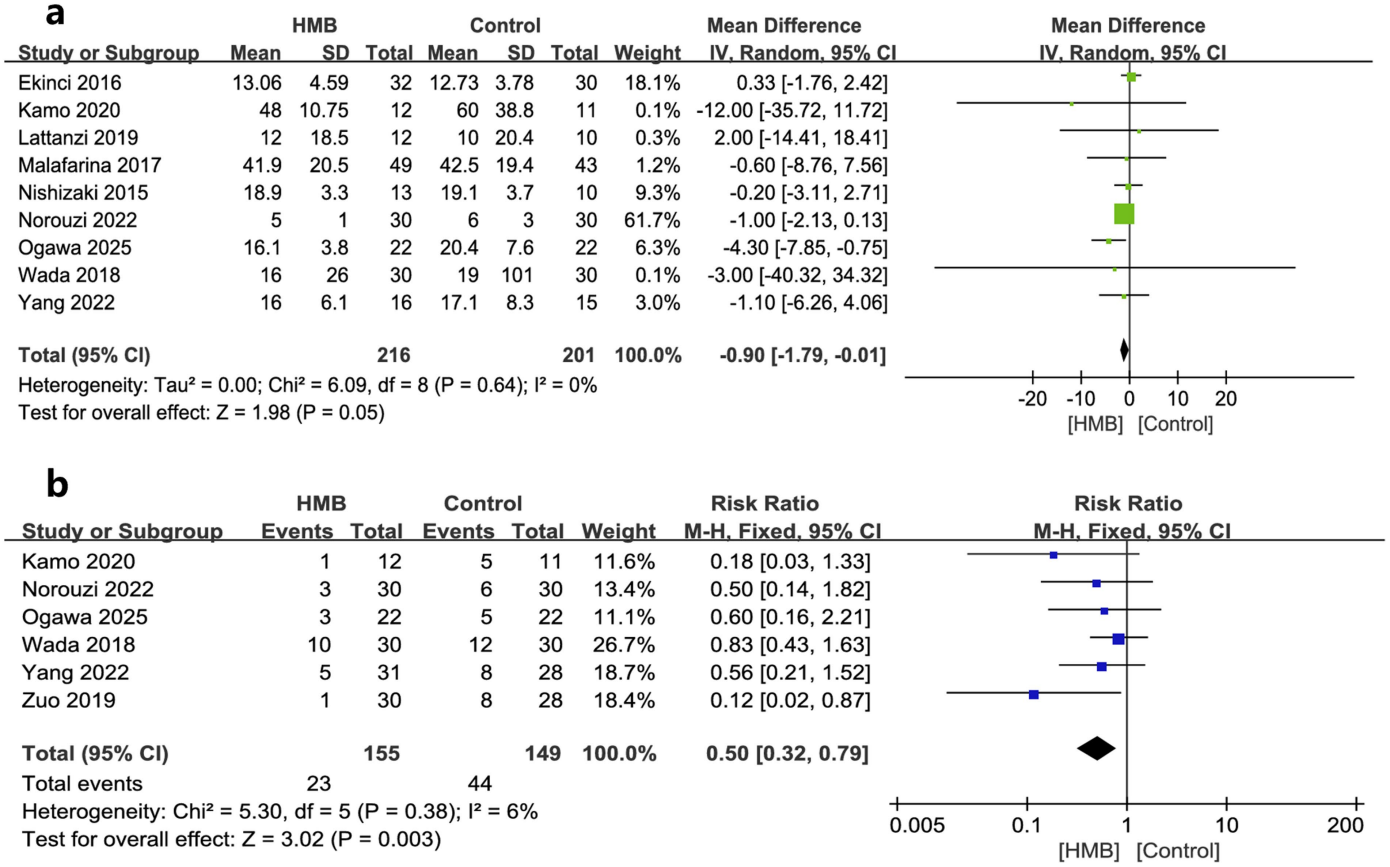
Forest plots of the beta-hydroxy-beta-methylbutyrate on length of stay in hospital **(a)** and postoperative complications **(b)** in surgical patients.

Hospital LOS and postoperative complications were reported in nine (18–20, 28–33) and seven (18–20, 27, 30, 33) RCTs, respectively. Our analyses showed that HMB significantly reduced hospital LOS (MD −0.90 days; 95% CI, −1.79 to −0.01; *I*^2^ = 0%, *p* = 0.05; Figure 2a) and decreased postoperative complications (RR 0.50; 95% CI, 0.32 to 0.79; *I*^2^ = 0%, *p* = 0.003; Figure 2b) compared with the control group. Although we found no statistical heterogeneity, we conducted stratified analyses based on predefined main study characteristics and clinical conditions to explore any additional source of heterogeneity. The sensitivity analysis, which excluded any single study, yielded results closely aligned with the overall combined estimates. For hospital LOS, the *p* values ranged from 0.02 to 0.36, with all *I*^2^ = 0%. For postoperative complications, the *p* values ranged from 0.003 to 0.05, with *I*^2^ ranging from 0 to 26%. Subgroup analyses were also conducted, and most pooled subgroup results supported the reduction of hospital LOS and complications in the HMB group (Table 3).

**Table 3:** Subgroup analyses of the effect of HMB on mortality in critically ill patients.

Study characteristics		References	Patient number	Mean difference/risk ratio (95% CI)	*I* ^2^	*p*-value
Length of stay in hospital		(18–20, 28–33)	417	−0.90 (−1.79, −0.01)	0	0.05
Exercise	With exercise	(18, 20, 31, 32)	182	−2 (−4.78, 0.78)	22%	0.16
	Without exercise	(19, 28–30, 33)	235	−0.70 (−1.684, 0.27)	0%	0.16
Design	Double-blind	(18, 19, 33)	143	−1.03 (−2.16, 0.10)	0%	0.07
	No double-blind	(20, 28–32)	274	−0.71 (−2.19, 0.778)	2%	0.34
Age	≥65 years	(20, 28, 30–33)	312	−0.72 (−2.18, 0.74)	1%	0.45
	<65 years	(18, 19, 29)	105	−1.01 (−2.14, 0.12)	0%	0.08
Protocol	HMB alone	(29–31)	145	−0.76 (−4.97, 3.45)	0%	0.72
	HMB combined other drugs	(18–20, 28, 32, 33)	272	−0.93 (−2.12, 0.26)	16%	0.13
Location	Asian study	(18, 20, 30, 32, 33)	181	1.75 (0.61, 4.98)	0%	0.08
	Non-Asian study	(19, 28, 29, 31)	236	−0.69 (−1.67, 0.30)	0%	0.17
Surgical site	Abdominal	(18, 29, 30, 33)	136	−1.31 (−6.09, 3.47)	0%	0.59
	Orthopedic	(27, 28, 31, 32)	177	0.12 (−1.54, 1.78)	0%	0.89
	Cardiac	(19, 20)	83	−2.20 (−5.32, 0.91)	67%	0.17
Postoperative complications		(18–20, 27, 30, 33)	304	0.50 (0.32, 0.79)	0%	0.003
Exercise	With exercise	(18, 20)	67	0.39 (0.13, 1.11)	0%	0.08
	Without exercise	(19, 27, 30, 33)	237	0.54 (0.33, 0.88)	22%	0.01
Design	Double-blind	(18, 19, 33)	143	0.60 (0.34, 1.06)	16%	0.08
	No double-blind	(20, 27, 30)	161	0.40 (0.20, 0.82)	12%	0.01
Age	≥65 years	(20, 27, 30, 33)	221	0.56 (0.34, 0.91)	20%	0.02
	<65 years	(18, 19)	83	0.35 (0.12, 1.02)	0%	0.05
Protocol	HMB alone	(27, 30)	117	0.34 (0.14, 0.82)	52%	0.02
	HMB combined other drugs	(18–20, 33)	187	0.60 (0.36, 1.01)	0%	0.05
Location	Asian study	(18, 20, 27, 30, 33)	244	0.51 (0.32, 0.81)	0%	0.005
	Non-Asian study	(19)	60	0.50 (0.14, 1.82)	—	0.29
Surgical site	Abdominal	(18, 30, 33)	142	0.61 (0.36, 1.04)	11%	0.07
	Orthopedic	(27)	58	0.12 (0.02, 0.87)	—	0.04
	Cardiac	(19, 20)	104	0.55 (0.22, 1.36)	0%	0.19

HMB, beta-hydroxy-beta-methyl butyrate.

### Secondary outcomes

**Figure 3:**
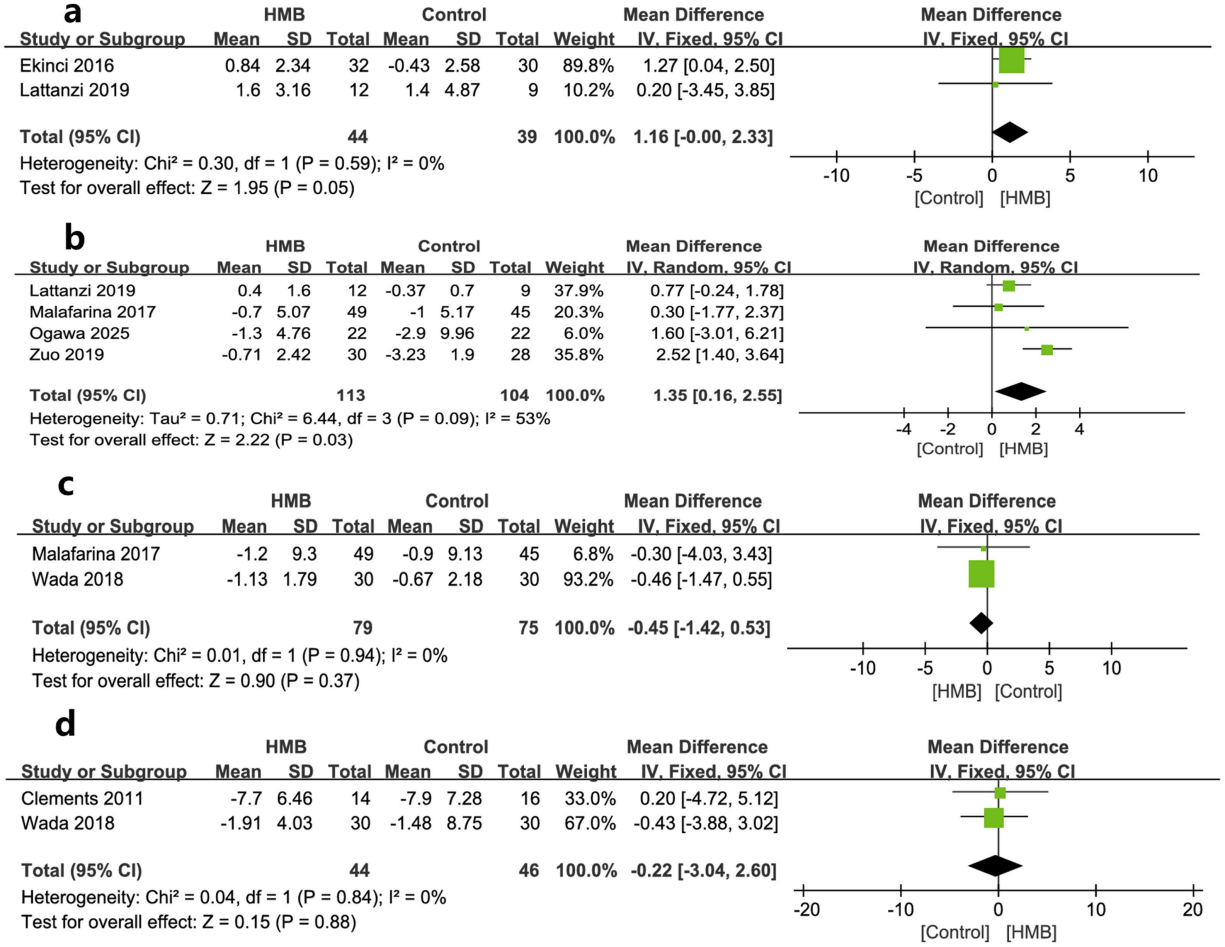
Forest plots of the p beta-hydroxy-beta-methylbutyrate on mid-arm muscle-circumference **(a)**, appendicular skeletal muscle mass **(b)**, skeletal muscle mass **(c)**, and lean body mass **(d)** in surgical patients.

As to muscle measures, MAMC, ASMM, SMM, and LBM were described by two, four, two, and two studies, respectively. The pooled estimates showed that compared with the control group, the HMB group had a more significant increase in changes regarding MAMC (MD 1.16 cm; 95% CI, 0 to 2.33; *p* = 0.05; Figure 3a) (28, 29) and ASMM (MD = 1.35 kg; 95% CI, 0.16–2.55; *p* = 0.03; Figure 3b) (20, 27, 29, 31), but had similar changes in SMM (MD = −0.45 kg; 95% CI, −1.42 to 0.53; *p* = 0.37; Figure 3c) (31, 33), and LBM (MD −0.22 kg; 95% CI, −3.04 to 2.60; *p* = 0.88; Figure 3d) (26, 33).

**Figure 4:**
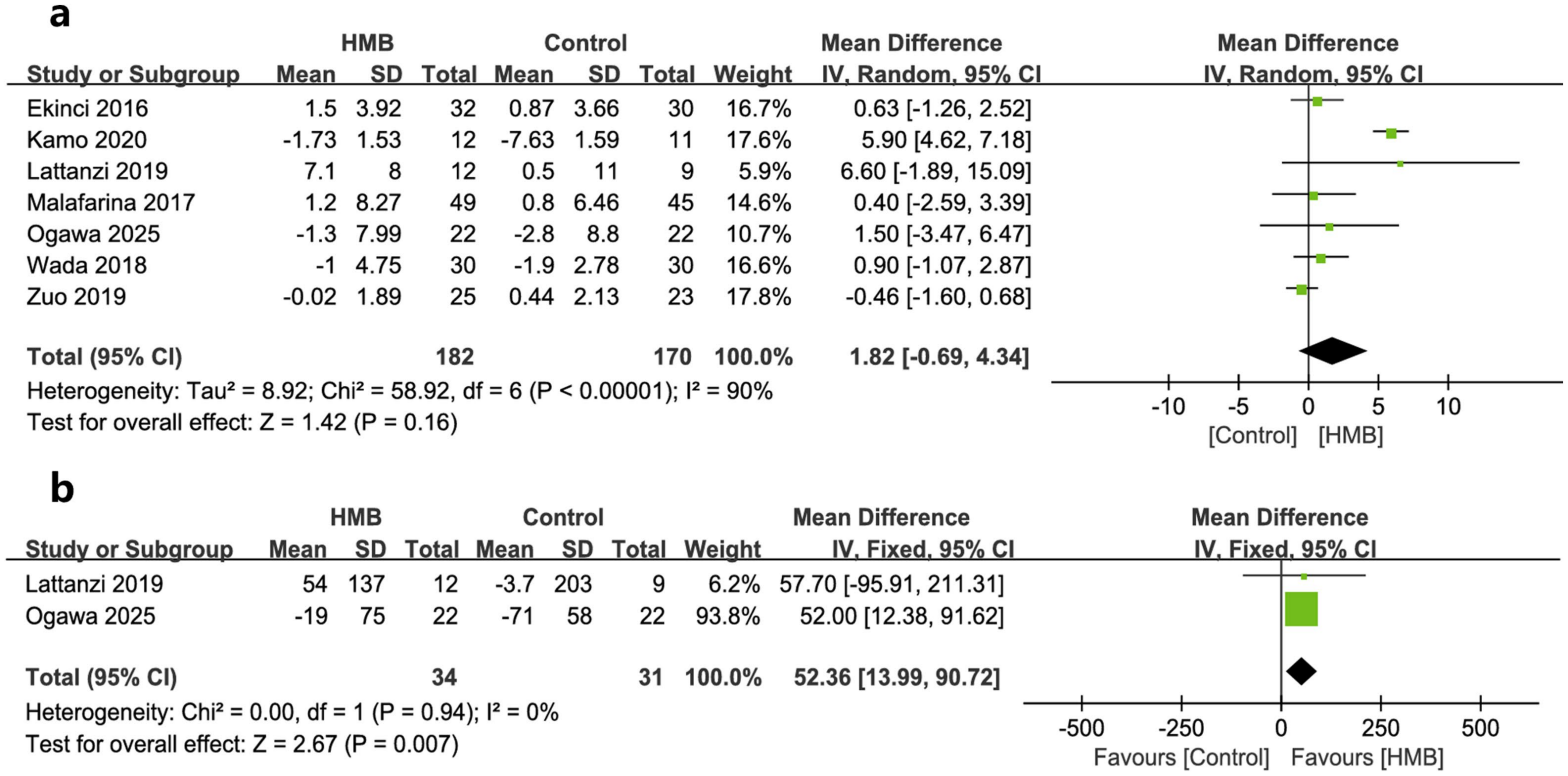
Forest plots of the p beta-hydroxy-beta-methylbutyrate on hand grip strength **(a)** and 6-min walking distance **(b)** in surgical patients.

As to outcomes of physical function, changes in HGS, 6-MWD, and GS was reported by seven, two, and one trials, respectively. When pooled, no significant differences were found in changes in HGS (MD = 1.82; 95% CI, −0.69 to 4.34; *p* = 0.16; Figure 4a) (18, 20, 27–29, 31, 33) between the two groups. However, patients in HMB had significantly more 6-MWD (MD = 52.36 m; 95% CI, 13.99 to 90.72; *p* = 0.007; Figure 4b) than control group. In addition, one study reported a significant improvement in GS than the controls (*p* = 0.0002) (20).

**Figure 5:**
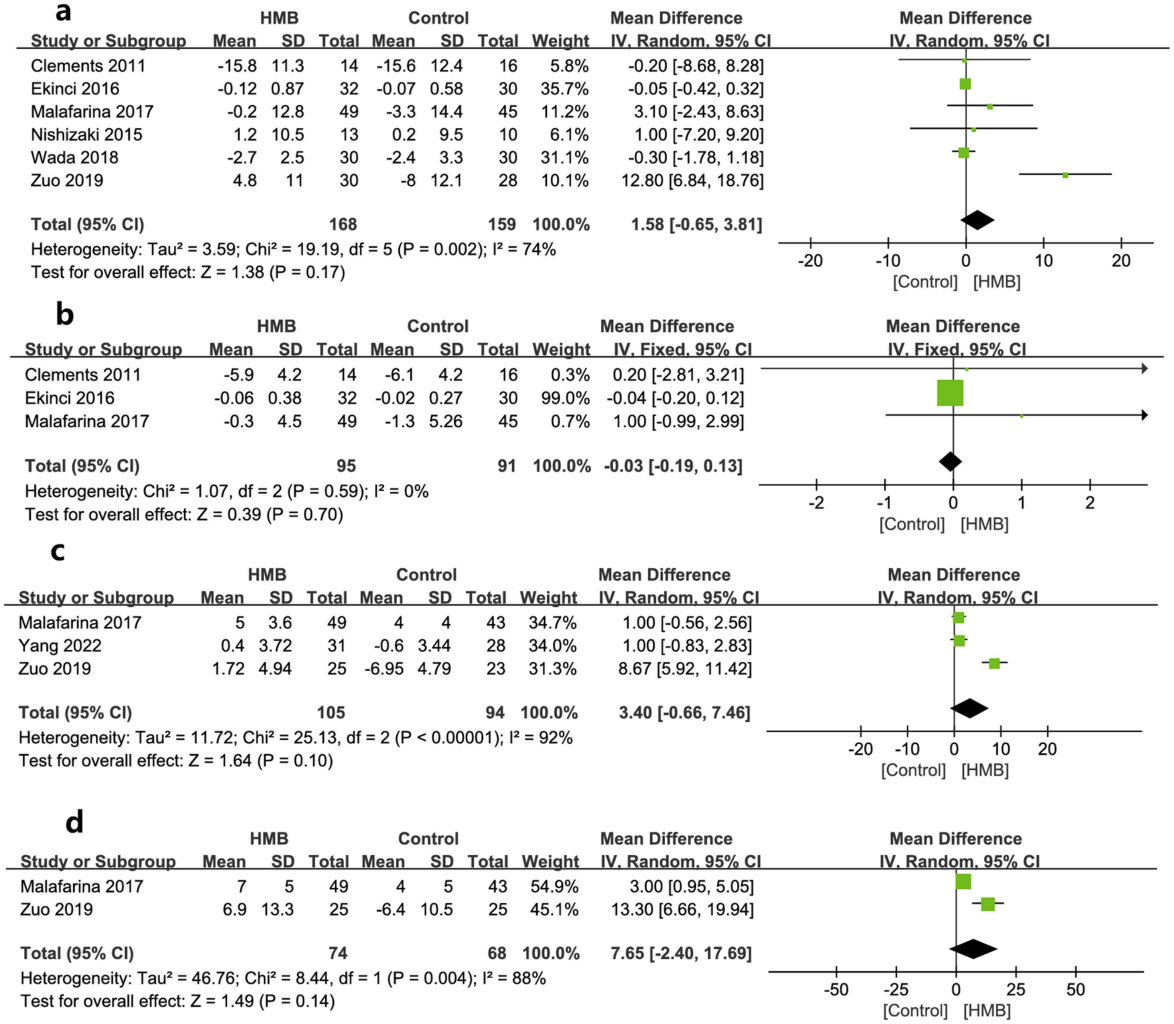
Forest plots of the p beta-hydroxy-beta-methylbutyrate on body weight **(a)**, body mass index **(b)**, serum albumin **(c)**, and total albumin **(d)** in surgical patients.

Regarding other variables of nutritional status, compared to the control group, the HMB group did not show significant improvement from baseline after treatment, including BW (MD = 0.21 kg; 95% CI, −0.15 to 0.58; *p* = 0.25; Figure 5a) (26–28, 31–33), BMI (MD = 0.07 kg/m^2^; 95% CI, −0.22 to 0.36; *p* = 0.63; Figure 5b) (26, 28, 31), serum albumin (MD = 3.40 g/L; 95% CI, −0.66 to 7.46; *p* = 0.10, Figure 5c) (27, 30, 31), and total albumin (MD = 7.65 days; 95% CI, −2.40 to 17.69; *p* = 0.14; Figure 5d) (27, 31).

## Discussion

Our study indicated that muscle loss occurs commonly in surgical patients. The current meta-analysis of 11 RCTs suggested that perioperative HMB supplementation improved patient outcomes. Specifically, HMB significantly reduced hospital LOS and postoperative complications. Additionally, pooled results from a few included RCTs suggested HMB provided significant benefits over conventional treatment in some muscle measurements and physical function, such as MACA, ASMM, and 6-MWD, but did not improve other parameters of nutrition status. As far as we know, this is the first meta-analysis to investigate the effects of supplementing with HMB or HMB-rich nutritional supplements in surgical patients.

### HMB technology research

For many years, HMB has been used in athletes for muscle building, strength, endurance enhancement (34, 35), and recovery after exercise-induced muscle injury (12). In recent years, its interest has rapidly expanded to include the elderly ill populations (15), sarcopenia (14), cancer (16), and critically ill patients (17). A meta-analysis suggested that 12 weeks of HMB supplementation improved muscle mass, strength, and physical function in the elderly population (15). Another meta-analysis that included nine RCTs suggested that HMB improved muscle mass and strength, but there was no evidence of benefits for physical function in patients with sarcopenia (14). Similarly, Prado et al. (16) pooled the results from 15 studies of cancer patients treated with HMB and showed that HMB had a beneficial effect on muscle mass and function in this patient population. These studies consistently demonstrated the beneficial effects of HMB in elderly or frail populations and were consistent with some of our findings. Conversely, in studies of critically ill patients, HMB did not improve mortality or other clinical outcomes in ICU patients (17). This ineffectiveness may be related to the HMB strategy used in the included trials. Our study included surgical patients who were also from a population with advanced age, tumors, and heart diseases and were subjected to varying degrees of stress from surgery. We analyzed the effects of HMB comprehensively on clinical outcomes, muscle strength, mass, body function, and nutrition in this population, initially showing the benefits of HMB and/or its additives. Our study adds a new population for clinical HMB application in terms of meta-analysis evidence.

### Interpretation of study results

Although HMB can benefit surgical patients, several issues are worth exploring. First, our primary outcomes were hospital LOS and postoperative complications, as they were the most selections among the included studies (18–20, 28–33). This selection reflected that clinicians pay more attention to patient-centered clinical outcomes. However, the effect of HMB on the outcome of hospital LOS is indirect. Meanwhile, hospital LOS is a relatively subjective outcome since it is often influenced by clinical practices such as bed availability, turnover, and patient wishes. Fortunately, the clinical aspect of the benefits is supported by the positive outcome findings of complications, muscle mass, and functional activity. Moreover, most subgroup analyses based on predefined influencing factors showed a tendency to benefit HMB supplements in surgical patients.

In contrast, the outcome of complications is relatively objective. Most of the complications reported in the included articles focused on nosocomial infections, including surgical site, lung, and urinary tract infections (19, 20, 27, 33). HMB has shown effects in promoting wound healing (36), lowering CPK (37), and increasing serum growth hormone levels (33). The latter may promote wound healing (38). Reducing these complications contributes to the success of the surgery and subsequent recovery.

Second, the effectiveness of HMB may be influenced by the strategy of its application. The previous meta-analysis of the critically ill population not benefiting from HMB may be related to ICU patients receiving HMB later (17). In the study by Supinski et al. (39), patients had received mechanical ventilation for an average of 6 days before HMB supplementation. Theoretically, HMB administered after muscle weakness did not improve muscle function. Therefore, this delay may have hindered the beneficial effects of HMB therapy. Meanwhile, ICU patients often suffer from gastrointestinal dysfunction, as well as fasting and gastric decompression (40), which may impair drug absorption and limit the effectiveness of HMB in improving muscle function. On the contrary, the timing of HMB administration in surgical patients can be initiated very early in the preoperative period, i.e., some studies have started patients on HMB as early as 10–30 days before surgery (19, 20, 30). Most patients can take HMB orally before surgery and get good absorption. Importantly, surgical patients are very receptive and compliant with HMB therapy. For example, compliance was as high as 95% in the study by Ogawa et al. (20). Moreover, some investigators have controlled and promoted compliance by asking patients treated with HMB to return the HMB/placebo bag the day before surgery (19). These differences may be why surgical patients benefit more from HMB than critically ill patients. In addition, rehabilitation performed preoperatively and postoperatively in surgical patients is easier to implement and works well with HMB (20, 32). Conversely, ICU patients who receive HMB rarely undergo rehabilitation. Even if they receive rehabilitation, the effect is not accurate. One ICU study reported that their patients were poorly trained (<10 min/day) (39). Therefore, using perioperative HMB, especially preoperatively, may be a promising area.

In addition, the beneficial effects of HMB may be influenced by the synergistic effect of common additives, including ARG and Glu (18–20, 26, 28, 32, 33). However, these included trials did not compare individuals and combinations, making it difficult to assess the effects of HMB alone. Some previous studies have suggested that ARG and Glu, as immune nutrients, can significantly improve the immunity and infection of postoperative patients (41, 42). However, Kuhls et al. (43) compared HMB alone or in combination with arginine and glutamine in trauma patients and found no difference between use alone and in combination, and HMB significantly improved nitrogen balance. In addition, a meta-analysis suggests that the presence of HMB may exacerbate the prognosis of ICU patients (44). Therefore, future studies need to elucidate the specific role of these amino acids and their optimal combination in perioperative nutritional support.

HMB improves muscle measurements and physical function and is thought to promote protein synthesis and inhibit protein catabolism *in vivo* through complex mechanisms (45, 46). Recently, clinical emphasis has been placed on the importance of adequate nutritional support combined with rehabilitation in muscle protein maintenance and synthesis (47). The included studies support this view. For example, the results of HMB improvement in ASMM were pooled from four RCTs (20, 27, 29, 31), all of which reflected an emphasis on adequate nutritional support (20, 29, 31) and the implementation of early rehabilitation (20, 31). Similarly, HMB improved 6-MWD by pooling results from two studies describing detailed nutrition programs and early rehabilitation (20, 29).

### Limitations

Our study has some limitations. First, most included RCTs were small-sample, open-label studies with potentially high selection bias. Second, only a few studies provided data for pooling in the secondary outcomes, which limited our implementation of subgroup analyses of these outcomes. Therefore, interpretation of these outcomes requires caution. Third, although there was no statistically significant heterogeneity in the primary outcomes, some potential clinical heterogeneity remained unresolved. For example, there were variations in the gender distribution and types of surgery among study participants. These differ substantially in physiological stress, postoperative recovery patterns, nutritional risk, and potential for muscle catabolism. Meanwhile, the included study’s definition of complications varied substantially, which depended on the surgical procedures. Fourth, since nearly half of the included RCTs administered HMB as a single supplement, more future studies should clarify the independent influencing role of HMB. Fifth, exploring the ideal timing (or regimen) of HMB preoperative supplementation remains unclear due to the limited availability of data from the included studies. Sixth, most studies provided results at different time points for assessments. Since many of the outcomes assessed (e.g., physical function, nutritional markers) are time-sensitive, these inconsistencies in measurement timing may influence the pooled effect estimates. Finally, some postoperative patients had ICU admissions. However, we could not evaluate the efficacy of these ICU patients separately due to insufficient data.

## Conclusion

Our analysis shows that HMB alone or its complexes significantly reduce length of stay in hospital and postoperative complications in surgical patients. Meanwhile, HMB improved MAMC, ASMM, and 6-MWD but did not improve other parameters of the nutrition status in this patient population. The limitations of the included studies are prominent, such as the study design, small sample size, and the high risk of bias, which may have contributed to the low certainty of our results. Future research should be well-designed to clarify the effects of HMB in surgical patients.

## Data Availability

The original contributions presented in the study are included in the article/Supplementary material, further inquiries can be directed to the corresponding author.
