# Supplementary material for: The effects of beta-hydroxy-beta-methyl butyrate supplementation in surgical patients: a systematic review and meta-analysis of randomized controlled trials
Source: Front Nutr. 2025 Jul 23;12:1621206. doi: 10.3389/fnut.2025.1621206 (PMC12326741; doi:10.3389/fnut.2025.1621206)
Supplement: Supplementary file 1 [file Supplementary_file_1.DOCX]

**Beta-hydroxy-beta-methylbutyrate supplementation in surgical patients: a systematic review and meta-analysis of randomized controlled trials**

**Additional Files**

Additional File 1 PRISMA checklist…………………………………………………………………………………………………………………………………………………………………………………………….…………………………………..…..2

Additional File 2 Search Strategy……………………………………………………………………………………………………………………………………………………………………………………………………………….……………………....5

Additional File 3 Studies needed for full-reviewed but not included in the current meta-analysis (n=6 trials) ……………………………………………………………………………………………..…………..7 Additional File 4 Assessment of RCTs quality …………….…………………………….…………………………………………………………………………………………………………………………………………………………….....….…….8

Additional File 5 Funnel plot of comparison: ……………………………….…..………………………………………………………………….…………………………………………………………….…………………………………....…………9

Additional File 6 Summary definitions of complications in each included study……………………………………………………………………………………………………………………………………………………………….10

Additional File 7 GRADE evaluation…………………………. …………….…………………………….…………………………………………………………………………………………………………………………….………………….....…….11

**Additional File 1**

**PRISMA 2009 checklist**

| **Section/topic** | **#** | **Checklist item** | **Reported on page #** |
| --- | --- | --- | --- |
| **TITLE** | | |  |
| Title | 1 | Identify the report as a systematic review, meta-analysis, or both. | 1 |
| **ABSTRACT** | | |  |
| Structured summary | 2 | Provide a structured summary including, as applicable: background; objectives; data sources; study eligibility criteria, participants, and interventions; study appraisal and synthesis methods; results; limitations; conclusions and implications of key findings; systematic review registration number. | 3 |
| **INTRODUCTION** | | |  |
| Rationale | 3 | Describe the rationale for the review in the context of what is already known. | 5 |
| Objectives | 4 | Provide an explicit statement of questions being addressed with reference to participants, interventions, comparisons, outcomes, and study design (PICOS). | 5-6 |
| **METHODS** | | |  |
| Protocol and registration | 5 | Indicate if a review protocol exists, if and where it can be accessed (e.g., Web address), and, if available, provide registration information including registration number. | 7 |
| Eligibility criteria | 6 | Specify study characteristics (e.g., PICOS, length of follow-up) and report characteristics (e.g., years considered, language, publication status) used as criteria for eligibility, giving rationale. | 7 |
| Information sources | 7 | Describe all information sources (e.g., databases with dates of coverage, contact with study authors to identify additional studies) in the search and date last searched. | 7 |
| Search | 8 | Present full electronic search strategy for at least one database, including any limits used, such that it could be repeated. | 7-8 and Additional File 2 |
| Study selection | 9 | State the process for selecting studies (i.e., screening, eligibility, included in systematic review, and, if applicable, included in the meta-analysis). | 7 |
| Data collection process | 10 | Describe method of data extraction from reports (e.g., piloted forms, independently, in duplicate) and any processes for obtaining and confirming data from investigators. | 7-8 |
| Data items | 11 | List and define all variables for which data were sought (e.g., PICOS, funding sources) and any assumptions and simplifications made. | 8 |
| Risk of bias in individual studies | 12 | Describe methods used for assessing risk of bias of individual studies (including specification of whether this was done at the study or outcome level), and how this information is to be used in any data synthesis. | 8 |
| Summary measures | 13 | State the principal summary measures (e.g., risk ratio, difference in means). | 8 |
| Synthesis of results | 14 | Describe the methods of handling data and combining results of studies, if done, including measures of consistency (e.g., I^2^) for each meta-analysis. | 9 |

| Risk of bias across studies | 15 | Specify any assessment of risk of bias that may affect the cumulative evidence (e.g., publication bias, selective reporting within studies). | 8 |
| --- | --- | --- | --- |
| Additional analyses | 16 | Describe methods of additional analyses (e.g., sensitivity or subgroup analyses, meta-regression), if done, indicating which were pre-specified. | 9 |
| **RESULTS** | | |  |
| Study selection | 17 | Give numbers of studies screened, assessed for eligibility, and included in the review, with reasons for exclusions at each stage, ideally with a flow diagram. | 10and Figure 1, Additional File 2 |
| Study characteristics | 18 | For each study, present characteristics for which data were extracted (e.g., study size, PICOS, follow-up period) and provide the citations. | 10  Table 1, |
| Risk of bias within studies | 19 | Present data on risk of bias of each study and, if available, any outcome level assessment (see item 12). | 10-11 |
| Results of individual studies | 20 | For all outcomes considered (benefits or harms), present, for each study: (a) simple summary data for each intervention group (b) effect estimates and confidence intervals, ideally with a forest plot. | 11 |
| Synthesis of results | 21 | Present results of each meta-analysis done, including confidence intervals and measures of consistency. | 11-12 |
| Risk of bias across studies | 22 | Present results of any assessment of risk of bias across studies (see Item 15). | Additional File 3 |
| Additional analysis | 23 | Give results of additional analyses, if done (e.g., sensitivity or subgroup analyses, meta-regression [see Item 16]). | 11-12  Additional File 4-5 |
| **DISCUSSION** | | |  |
| Summary of evidence | 24 | Summarize the main findings including the strength of evidence for each main outcome; consider their relevance to key groups (e.g., healthcare providers, users, and policy makers). | 13-17 |
| Limitations | 25 | Discuss limitations at study and outcome level (e.g., risk of bias), and at review-level (e.g., incomplete retrieval of identified research, reporting bias). | 17 |
| Conclusions | 26 | Provide a general interpretation of the results in the context of other evidence, and implications for future research. | 18 |
| **FUNDING** | | |  |
| Funding | 27 | Describe sources of funding for the systematic review and other support (e.g., supply of data); role of funders for the systematic review. | 19 |

**Additional File 2**

**Search Strategy**

Database: 5 data bases

Search completed May 1st, 2024

----------------------------------------------------------------------------------------------------------------------

**PubMed**

1#.

“operation” OR “operative” OR “operated” OR “post-operative” OR “postoperative” OR “peri-operative” OR “perioperative” OR “surgical” OR “surgery” OR “surgeries”

2#.

(((beta hydroxy beta methyl butyrate[Title/Abstract]) OR (beta-hydroxy-beta-methylbutyric acid[Title/Abstract])) OR (β-hydroxy-β-methylbutyrate[Title/Abstract])) OR (β-hydroxy-β-methyl butyrate[Title/Abstract])

3.

(randomized controlled trial [pt] OR controlled clinical trial [pt] OR randomized [tiab] OR placebo [tiab] OR clinical trials as topic [mesh: noexp] OR randomly [tiab] OR trial [ti]) NOT (animals [mh] NOT humans [mh])

4.

1# AND 2# AND 3#

**Embase**

No. Query Results

#18. #5 AND #13 AND #17

#17. #14 OR #15 OR #16

#16. random*:ab,ti AND [embase]/lim

#15. blind*:ab,ti AND [embase]/lim

#14. placebo:ab,ti AND [embase]/lim

#13. #6 OR #7 OR #8 OR #9 OR #10 OR #11 OR #12

#12. 'operation':ab,ti

#11. 'surgical':ab,ti

#10. 'peri-operative':ab,ti

#9. 'postoperative':ab,ti

#8. 'operated':ab,ti

#7. 'operative':ab,ti

#6. 'surgery':ab,ti

#5. #1 OR #2 OR #3 OR #4

#4. 'β-hydroxy-β-methyl butyrate':ab,ti

#3. 'β-hydroxy-β-methylbutyrate':ab,ti

#2. 'beta-hydroxy-beta-methylbutyric acid':ab,ti

#1. 'beta hydroxy beta methyl butyrate':ab,ti

**Cochrane library**

ID Search

#1 ("beta hydroxy beta methyl butyrate "):ti,ab,kw (Word variations have been searched)

#2 ("beta-hydroxy-beta-methylbutyric acid "):ti,ab,kw (Word variations have been searched)

#3 ("β-hydroxy-β-methylbutyrate "):ti,ab,kw (Word variations have been searched)

#4 ("β-hydroxy-β-methyl butyrate "):ti,ab,kw (Word variations have been searched)

#5 #1 OR #2 OR #3 OR #4

#6 ("surgery"):ti,ab,kw (Word variations have been searched)

#7 ("operative"):ti,ab,kw (Word variations have been searched)

#8 ("operated"):ti,ab,kw (Word variations have been searched)

#9 ("postoperative"):ti,ab,kw (Word variations have been searched)

#10 ("peri-operative"):ti,ab,kw (Word variations have been searched)

#11 ("surgical"):ti,ab,kw (Word variations have been searched)

#12 ("operation"):ti,ab,kw (Word variations have been searched)

#13 #6 OR #7 OR #8 OR #9 OR #10 OR #11 OR #12

#14 #5 AND #14

**Wanfang database**

主题：（外科 or 手术or 术后 or切除术 or 移植）and 主题：（β-羟基-β-甲基丁酸盐）

**China National Knowledge Infrastructure database**

TKA=“β-羟基-β-甲基丁酸盐” and TKA=("外科 + “手术” + “术后” + “切除术” + “移植”）

**Additional File 3**

**Table. Studies needed for full-reviewed but not included in the current meta-analysis (n=6 trials)**

| No | Study | Reason of exclusion |
| --- | --- | --- |
| 1 | Ferreira SC, Cardoso ASR, Machado AAS, Anastácio LR. Effect of a 12-week nutritional intervention in the food intake of patients on the waiting list for liver transplantation: A secondary analysis of a randomized controlled trial. Clin Nutr. 2024 Jun;43(6):1278-1290. | Included patients did not received operation |
| 2 | Wittholz K, Fetterplace K, Karahalios A, Ali Abdelhamid Y, Beach L, Read D, Koopman R, Presneill JJ, Deane AM. Beta-hydroxy-beta-methylbutyrate supplementation and functional outcomes in multitrauma patients: A pilot randomized controlled trial. JPEN J Parenter Enteral Nutr. 2023 Nov;47(8):983-992. | Included patients of trauma |
| 3 | Han Z, Ji NN, Ma JX, Dong Q, Ma XL. Effect of Resistance Training Combined with Beta-Hydroxy-Beta-Methylbutyric Acid Supplements in Elderly Patients with Sarcopenia after Hip Replacement. Orthop Surg. 2022 Apr;14(4):704-713. | Not RCT |
| 4 | Norouzi M, Nadjarzadeh A, Maleki M, Khayyatzadeh SS, Hosseini S, Yaseri M, Fattahi H. The effects of preoperative supplementation with a combination of beta-hydroxy-beta-methylbutyrate, arginine, and glutamine on inflammatory and hematological markers of patients with heart surgery: a randomized controlled trial. BMC Surg. 2022 Feb 11;22(1):51. | Duplicate study |
| 5 | Kanda M, Koike M, Fukaya M, Miyata K, Tanaka C, Kobayashi D, Hayashi M, Yamada S, Nakayama G, Murotani K, Fujiwara M, Nagino M, Kodera Y. A prospective trial to evaluate treatment effects of a β-hydroxy-β-methylbutyrate containing nutrient for leakage at the anastomotic site after esophagectomy. Nagoya J Med Sci. 2020 Feb;82(1):33-37. | Not RCT |
| 6 | Takebayashi K, Kaida S, Otake R, Fukuo A, Miyake T, Kojima M, Tani S, Maehira H, Mori H, Ishikawa H, Tani M. HMB/Arg/Gln may improve short-term outcomes after esophagectomy in patients with thoracic esophageal cancer. Dis Esophagus. 2025 Jan 7;38(1):doae121. | Not RCT |

**Additional File 4**

**Assessment of study quality**


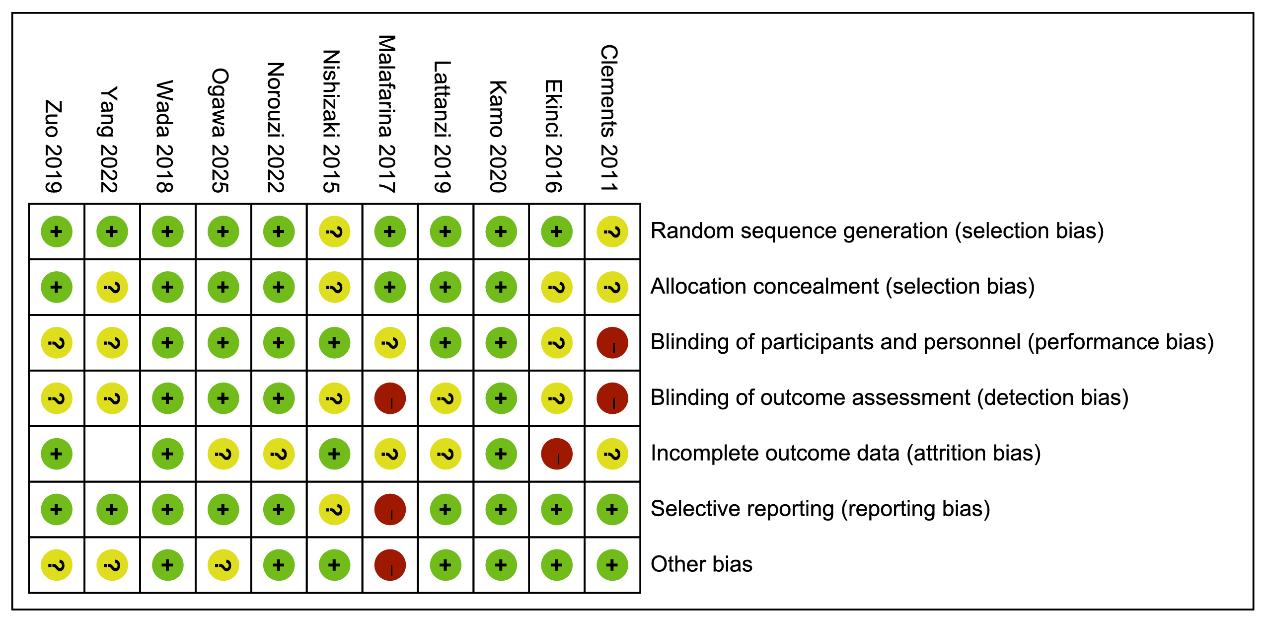


**Figure S1. Risk of bias graph: review authors' judgements about each risk of bias item presented as percentages across all included studies.**


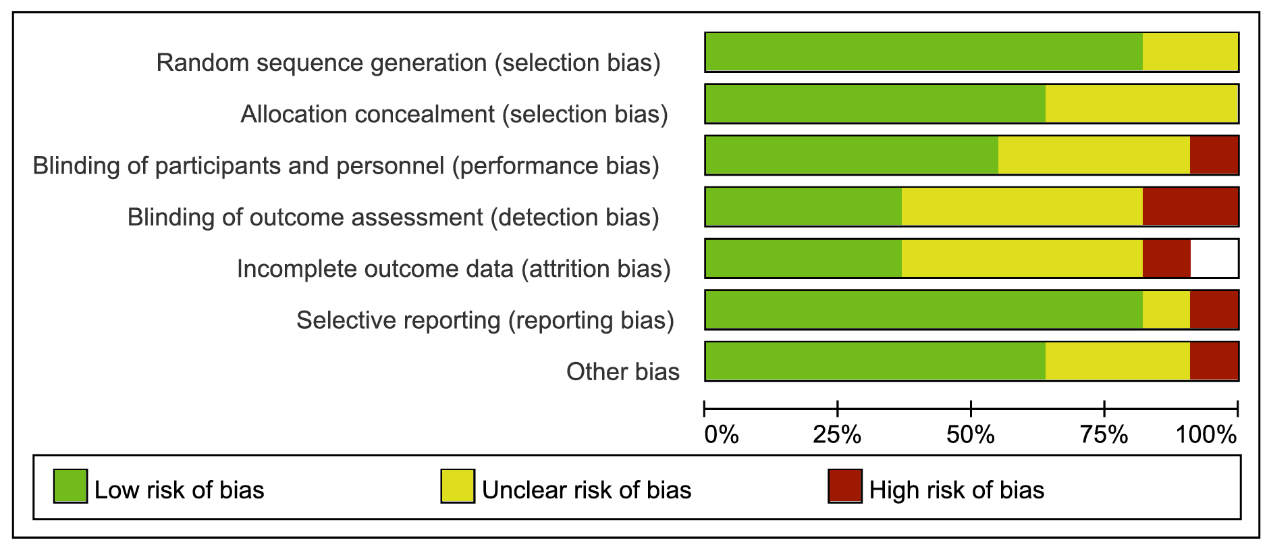


**Figure S2. Risk of bias summary: review authors' judgements about each risk of bias item for each included study**

**Additional File 5**

**Funnel plot of comparison: Length of stay in hospital**


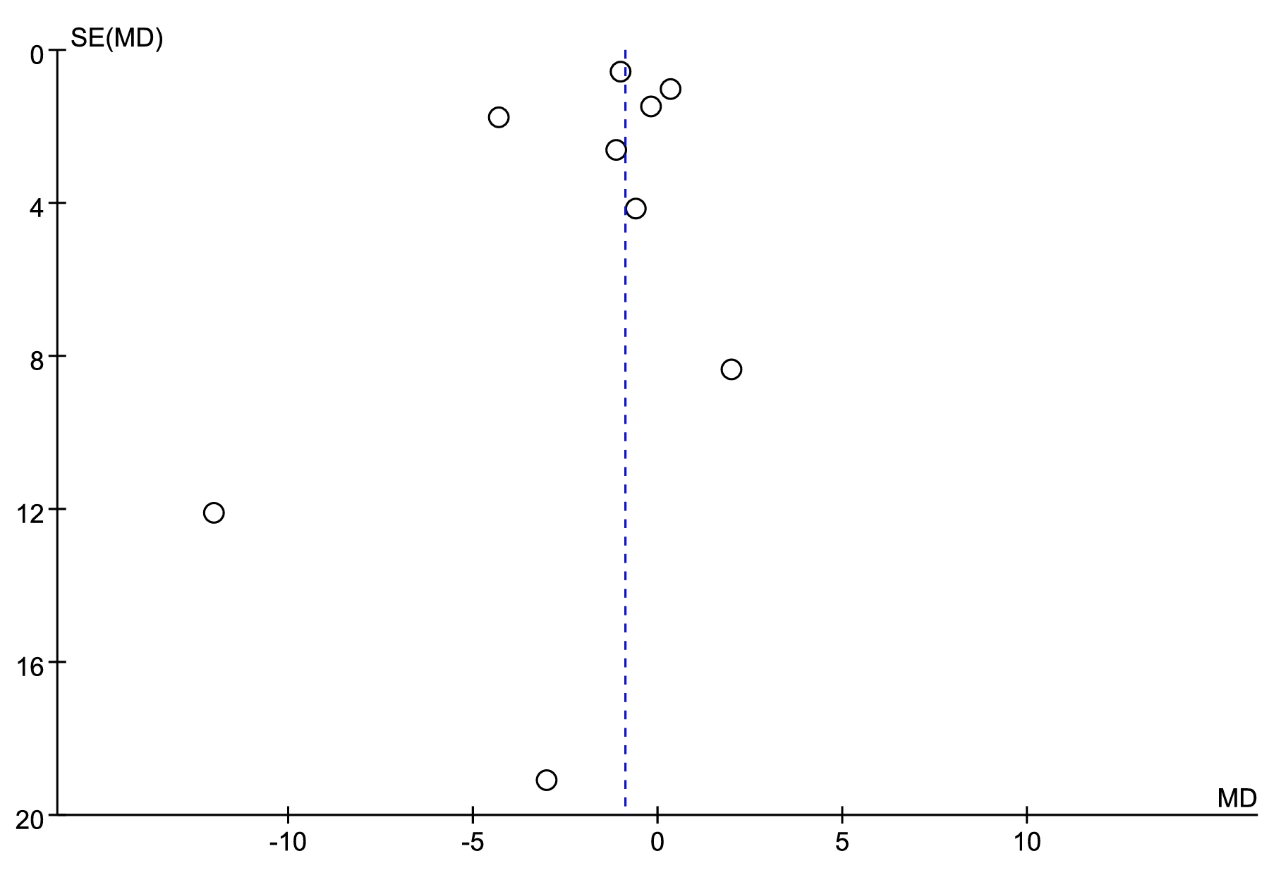


**Funnel plot of comparison: Postoperative complications**


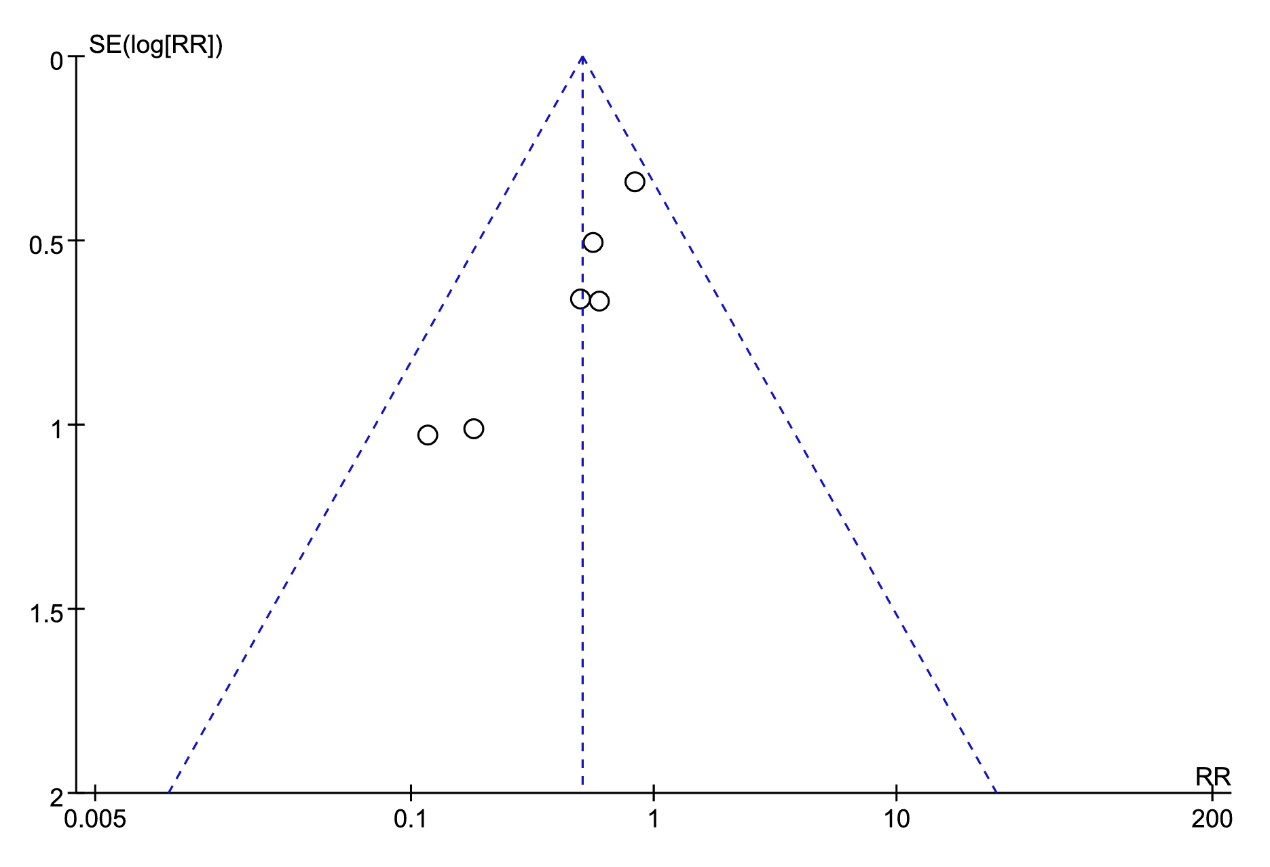


**Additional File 6:**

**Summary definitions of complications in each included study.**

| **Study** | **Definition of complication or Adverse events** | **Complication reported in studies** | **HMB** | **Control** |
| --- | --- | --- | --- | --- |
| Kamo 2020 | NA | **Incidence of bacteremia** | 1/12 | 8/11 |
| Norouzi 2022 | “Complications after surgery included the occurrence of myocardial infarction, stroke, acute kidney injury, atrial fibrillation, coma, and acute respiratory distress syndrome…” | **Complications after surgery** | 3/30 | 6/30 |
| Ogawa 2025 | NA | **In-hospital complications** | 3/22 | 5/22 |
| Wada 2018 | “…wound complications, defined as seroma, superficial incisional SSI, and wound dehiscence.” | **Wound complications** | 6/22 | 6/22 |
|  |  | Seroma | 4/22 | 4/22 |
|  |  | Superficial  incisional SSI | 2/22 | 2/22 |
|  |  | Wound dehiscence | 0/22 | 0/22 |
|  | “The severity of postoperative complications (excluding wound complications) was evaluated according to the Clavien-Dindo classification system” | **Any complications (≥ grade III)** | 4/22 | 6/22 |
|  |  | Pancreatic fistula | 1/22 | 2/22 |
|  |  | Leakage | 1/22 | 1/22 |
|  |  | Abdominal  abscess | 1/22 | 1/22 |
|  |  | Others | 1/22 | 2/22 |
| Yang 2022 | NA | **Complications** | 5/31 | 8/28 |
| Zuo 2019 | NA | **Complications** | 1/30 | 8/28 |

**Additional File 7: GRADE** **profile.**

**Bibliography:** . HMB for. Cochrane Database of Systematic Reviews [Year], Issue [Issue].

| **Quality assessment** | | | | | | | **Summary of findings** | | | | | **Importance** |
| --- | --- | --- | --- | --- | --- | --- | --- | --- | --- | --- | --- | --- |
|  |  |  |  |  |  |  | **No of patients** | | **Effect** | | **Quality** |  |
| **No of studies** | **Design** | **Limitations** | **Inconsistency** | **Indirectness** | **Imprecision** | **Other considerations** | **HMB** | **Control** | **Relative (95% CI)** | **Absolute** |  |  |
| **Hospital LOS (follow-up mean 20 days; Better indicated by lower values)** | | | | | | | | | | | | |
| 9 | randomised trials | serious^1^ | no serious inconsistency | no serious indirectness | no serious imprecision | none | 216 | 201 | - | MD 0.9 lower (1.79 to 0.01 lower) | ⊕⊕⊕O MODERATE | CRITICAL |
| **Complications** | | | | | | | | | | | | |
| 6 | randomised trials | serious^1^ | no serious inconsistency | no serious indirectness | no serious imprecision^1^ | none | 23/155 (14.8%) | 44/149 (29.5%) | RR 0.5 (0.32 to 0.79) | 148 fewer per 1000 (from 62 fewer to 201 fewer) | ⊕⊕⊕O MODERATE | CRITICAL |
|  |  |  |  |  |  |  |  | 28.6% |  | 143 fewer per 1000 (from 60 fewer to 194 fewer) |  |  |
| **HG (Better indicated by lower values)** | | | | | | | | | | | | |
| 7 | randomised trials | no serious limitations | serious^1^ | no serious indirectness | serious^1^ | none | 182 | 170 | - | MD 1.82 higher (0.69 lower to 4.34 higher) | ⊕⊕OO LOW | CRITICAL |
| **BW (Better indicated by lower values)** | | | | | | | | | | | | |
| 6 | randomised trials | serious^1^ | serious^1^ | no serious indirectness^1^ | serious^1^ | none | 168 | 159 | - | MD 1.58 higher (0.65 lower to 3.81 higher) | ⊕OOO VERY LOW | IMPORTANT |
| **BMI (Better indicated by lower values)** | | | | | | | | | | | | |
| 3 | randomised trials | serious^1^ | no serious inconsistency | no serious indirectness | very serious^1^ | none | 95 | 91 | - | MD 0.03 lower (0.19 lower to 0.13 higher) | ⊕OOO VERY LOW | IMPORTANT |
| **Albumin (Better indicated by lower values)** | | | | | | | | | | | | |
| 3 | randomised trials | serious^1^ | serious^1^ | no serious indirectness | serious^1^ | none | 105 | 94 | - | MD 3.4 higher (0.66 lower to 7.46 higher) | ⊕OOO VERY LOW |  |
| **ASMM (Better indicated by lower values)** | | | | | | | | | | | | |
| 4 | randomised trials | no serious limitations | serious^1^ | no serious indirectness | serious^1^ | none | 113 | 104 | - | MD 1.35 higher (0.16 to 2.55 higher) | ⊕⊕OO LOW | CRITICAL |
| **SMM (Better indicated by lower values)** | | | | | | | | | | | | |
| 2 | randomised trials | serious^1^ | no serious inconsistency | no serious indirectness | serious^1^ | none | 79 | 75 | - | MD 0.45 lower (1.42 lower to 0.53 higher) | ⊕⊕OO LOW |  |
| **6 MWD (Better indicated by lower values)** | | | | | | | | | | | | |
| 2 | randomised trials | serious^1^ | no serious inconsistency | no serious indirectness | serious^1^ | none | 34 | 31 | - | MD 52.36 higher (13.99 to 90.72 higher) | ⊕⊕OO LOW |  |

^1^ No explanation was provided

GRADE Working Group grades of evidence

- High quality: Further research is very unlikely to change our confidence in the estimate of effect.

- Moderate quality: Further research is likely to have an important impact on our confidence in the estimate of effect and may change the estimate.

- Low quality: Further research is very likely to have an important impact on our confidence in the estimate of effect and is likely to change the estimate.

- Very low quality: We are very uncertain about the estimate.
